# Acute Respiratory Distress in a Hospitalized Patient Revealing Cervical Diffuse Idiopathic Skeletal Hyperostosis (DISH)

**DOI:** 10.7759/cureus.87042

**Published:** 2025-06-30

**Authors:** Manish Kumar, Sudip Saha, Imtiaz A Khan, Sangita Kamath, Ashok Sunder

**Affiliations:** 1 General Medicine, Tata Main Hospital, Jamshedpur, IND

**Keywords:** dysphagia, osteophyte, skeletal hyperostosis, spine, stridor

## Abstract

Diffuse idiopathic skeletal hyperostosis (DISH) is a clinical condition marked by calcification and ossification of primarily ligaments of the spine and peripheral entheses. It predominantly affects older adults, with a prevalence rate of up to 42% in individuals over 65 years. It commonly presents with dysphagia. Stridor secondary to osteophyte compression has rarely been documented. This report discusses a patient who was admitted for alcohol intoxication following binge drinking and subsequently developed respiratory distress and unexplained stridor during hospitalization. He was intubated twice and later diagnosed with cervical diffuse idiopathic skeletal hyperostosis (DISH). The patient was discharged with a tracheostomy tube in situ and referred to a higher center for surgical management.

## Introduction

The understanding of diffuse idiopathic skeletal hyperostosis (DISH) has developed gradually over the last century, evolving from incidental pathological findings to a well-defined radiographic and clinical syndrome. The first known descriptions of widespread spinal ossification can be traced to early autopsy studies in the 19th century, where extensive bony overgrowth along the spine was occasionally noted but not well understood or classified [1]. These cases were often lumped together with other causes of spinal ankylosis, particularly ankylosing spondylitis (AS), due to similar appearances of spinal rigidity [2].

A pivotal contribution came in 1950, when Jacques Forestier and Jaume Rotes-Querol published a paper in which they described a condition characterized by flowing ossification of the anterior longitudinal ligament of the spine in elderly patients without sacroiliac joint involvement-distinguishing it from ankylosing spondylitis [2]. They referred to it as “senile ankylosing hyperostosis of the spine,” later colloquially known as Forestier’s disease.

In the 1970s, the term DISH was proposed by Resnick et al., who standardized the terminology and diagnostic criteria. Their radiographic criteria-flowing calcification and ossification along the anterolateral aspect of at least four contiguous vertebral bodies without significant intervertebral disc narrowing or sacroiliac joint changes-are still widely used today [3].

DISH is a non-inflammatory skeletal disease characterized by the formation of osteophytes, progressive ligamentous ossification, and preservation of the intravertebral disc space [4]. It is considered a systemic condition, and the exact cause is unclear but is often linked to aging and comorbid conditions such as diabetes mellitus, obesity, gout, and metabolic syndrome [5]. It typically involves the middle and lower thoracic spine, but can also involve other areas of the spine. These osteophytes may cause compression over the adjoining structures depending on their location. Osteophytes over lower cervical vertebrae (fourth, fifth, and sixth) can impinge on the oesophagus at the level of the cricoid cartilage, while those in the upper cervical spine can impinge on the oropharynx, causing globus, stridor, or respiratory compromise [6]. Osteophytes larger than 12 mm are likely to cause symptoms. Dysphagia as a common symptom of cervical osteophytes is described in the literature, but stridor secondary to osteophyte compression has rarely been documented [7].

We report a rare case of acute ethanol intoxication that developed acute respiratory distress with stridor and, on evaluation, was found to have cervical DISH with cervical osteophytes compressing the posterior larynx.

## Case presentation

A 63-year-old obese male patient was brought to Tata Main Hospital by his neighbours with altered sensorium following binge alcohol consumption. He had a BMI of 35, was hypertensive, and had a pacemaker in situ for complete heart block. On admission, he was afebrile with a Glasgow Coma Scale (GCS) of 10/15, blood pressure of 134/78 mmHg, pulse rate of 104 bpm, respiratory rate of 20/minute, and SpO2 on room air of 95%. There was no cyanosis, pallor, icterus, clubbing, or lymphadenopathy. He had a very short neck with restricted flexion and extension, but plantar reflexes were non-painful and bilaterally flexor. Systemic examination was normal. ECG on admission showed sinus tachycardia. A primary diagnosis of acute ethanol intoxication was made, and gastric lavage was performed. Symptomatic treatment with thiamine 100 mg intravenous (IV) thrice daily, 10% dextrose, IV lorazepam 1 mg thrice daily, and IV pantoprazole 40 mg was started.

The following morning, his sensorium improved, but he complained of breathlessness. Physical examination showed inspiratory stridor, increased respiratory rate with use of accessory muscles of respiration, and a decrease in oxygen saturation to 68% on ambient air. He was intubated and shifted to the critical care unit (CCU) for mechanical ventilation. He underwent a thorough evaluation for the cause of stridor, including radiograph of chest, blood gas analysis (pH, 7.39; HCO_3_, 23 mEq/L; PCO_2_, 42 mmHg), echocardiography, and contrast-enhanced computerised tomography (CECT) of chest. However, all results were within normal limits, and no evidence of pulmonary embolism was found. A direct laryngoscopy showed mild oedema of the posterior larynx.

**Figure 1:**
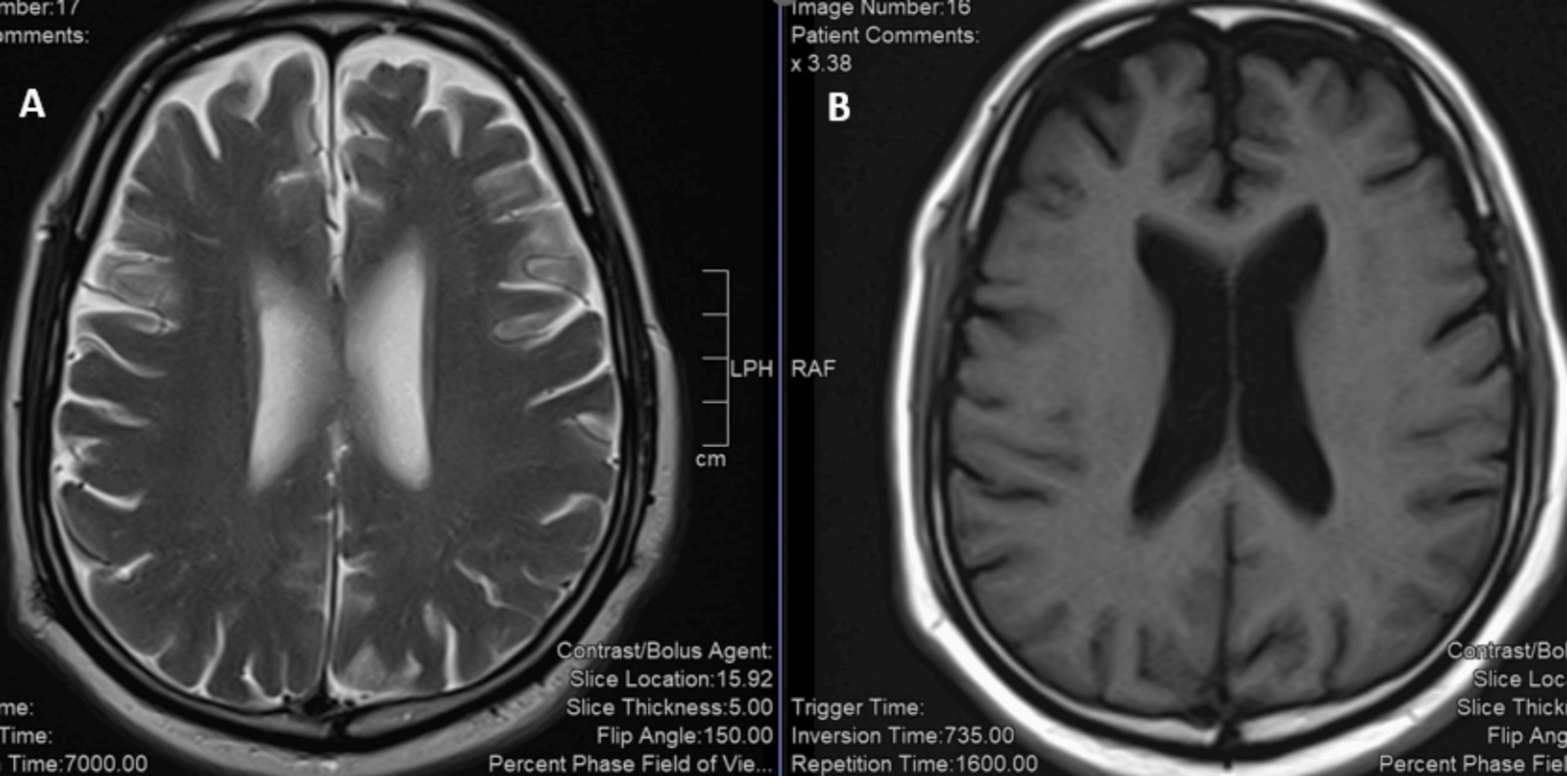
(A) T2-weighted and (B) T1-weighted imaging shows normal brain structures and signal intensities.

**Figure 2:**
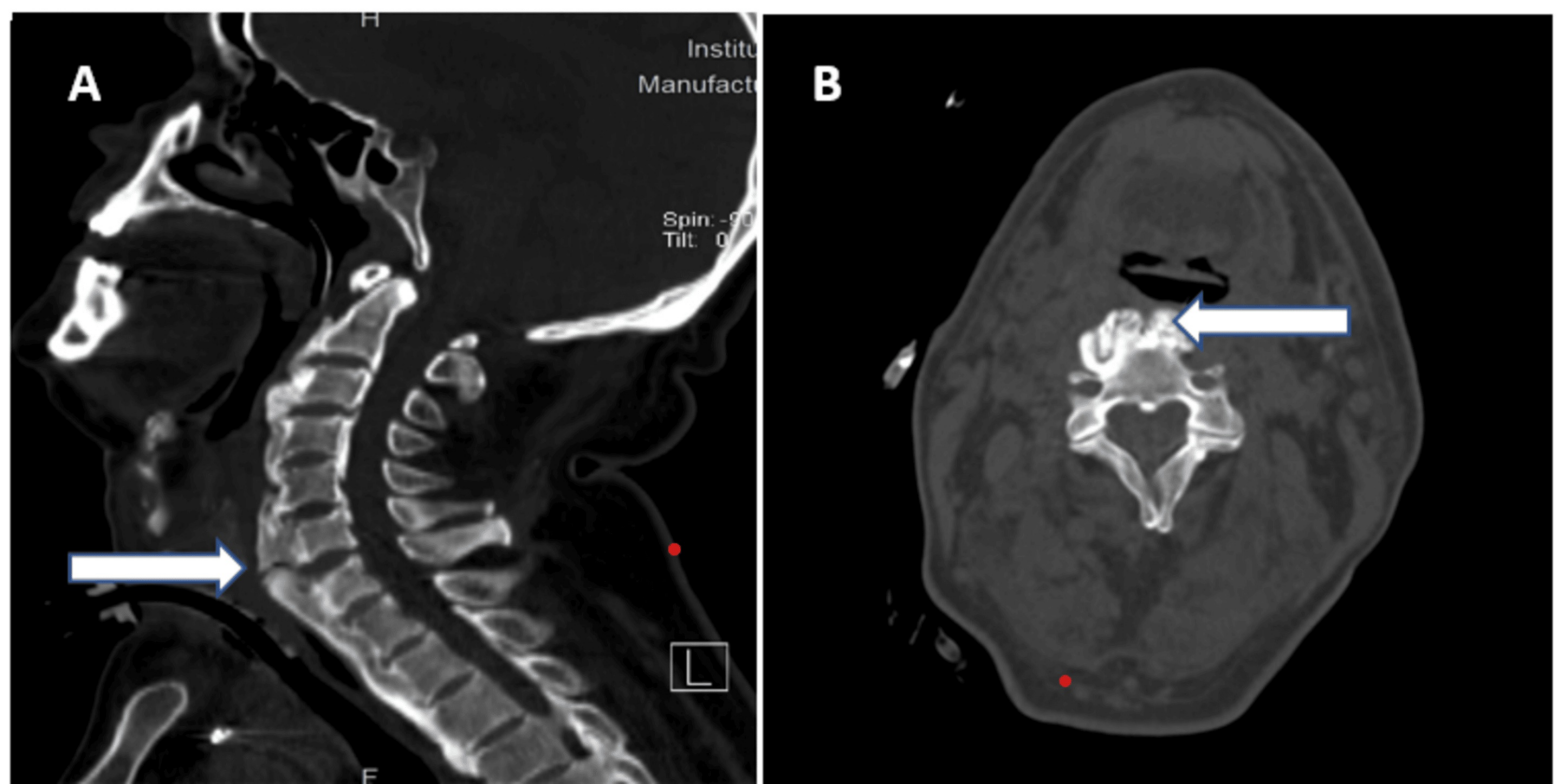
Sagittal (A) and axial (B) non-contrast CT scans in bone window of the cervical spine. White arrow shows exuberant and bulging anterior-bridging osteophytes causing indentation of the prevertebral space.

On the second day of reintubation, we noticed there was severe restriction of the neck movements. A CT scan of the neck and cervical spine (Figure 2A-2B) revealed ossification of the anterior longitudinal ligament (ALL) and osteophyte formation at the cervical and thoracic levels, suggestive of DISH.

These changes caused narrowing of the aero-pharyngeal tract. Due to airway narrowing, tracheotomy was performed. MRI of the sacro-iliac joints was normal, and HLA-B27 testing was negative, which ruled out ankylosing spondylitis. The patient was comfortable and asymptomatic post-tracheostomy without recurrence of stridor. Restriction of neck movement also improved to some extent with physiotherapy. Eventually, the patient was referred to a spinal center for definitive surgical management of DISH.

## Discussion

DISH, also known as Forestier disease, is an underdiagnosed disease with a prevalence of up to 42% in cases above 65 years of age [4]. It is more frequent in males and characterized by ligament ossification and entheses of the spine and peripheral skeleton [8]. A 2017 study indicated that DISH more commonly affects the anterior and lateral aspects of the spine with mild or no impact on mobility [9]. Diagnosis is made based on the radiological criteria of presence of ossification along at least four contiguous vertebrae, preservation of disc height without significant degenerative changes in the affected vertebral segments, and absence of apophyseal joint erosion, sclerosis, or ankylosis at the facet joints [9]. Anteroposterior and lateral spine radiographs typically show the characteristic "flowing candle wax" appearance, with ossification extending horizontally across vertebrae [10]. Since the thoracic spine is most commonly involved, radiographs should include this area. Differential diagnosis on anterior osteophytes includes degenerative cervical spine disease, like cervical spondylosis, and inflammatory arthritis like ankylosing spondylitis [10].

Most patients are asymptomatic or present with mild neck or back pain, with or without stiffness. However, cervical osteophytes can lead to airway obstruction, dysphagia, globus, bronchial aspiration, and hoarseness of voice. Cervical ossification can lead to life-threatening complications like stridor from respiratory obstruction. As the oesophagus is usually tethered at the level of cricoid cartilage between C4 and C6, most patients with lower cervical osteophytes present with dysphagia in 6% to 28% of the cases [11]. Similarly, in cases of laryngeal obstruction, in addition to the direct mechanical effect of osteophytes, secondary changes such as inflammatory oedema from repetitive mechanical trauma and ulceration may lead to fibrosis, formation of adhesions, and tethering of the vocal cords. Also, recurrent laryngeal nerve palsy due to compression by osteophytes contributes to respiratory obstruction [12].

Management typically includes activity modification, analgesics (NSAIDs), steroids, physical therapy, bracing, while surgical decompression is reserved for severe cases such as cervical myelopathy or persisting symptoms, with mortality being as high as 15%, higher than with conservative management [10].

Our patient had upper cervical osteophytes but had remained asymptomatic till admission. Perhaps aspiration, which may have occurred during alcohol intoxication, resulted in laryngeal oedema and tipped the balance. The reason cervical osteophytes, though frequently observed, often remain asymptomatic is not well understood. In a retrospective study by Nelson et al., involving 134 patients with DISH, it was observed that 3.8% of patients presented with dysphagia, and three patients required tracheotomy for acute airway obstruction due to osteophyte formation [13]. This should be considered as a possible aetiology of stridor in elderly individuals when other causes are ruled out.

A case report by Zarei et al. describes a 68‑year‑old man with a history of progressive dysphagia and recent dysphonia, and stridor secondary to cervical osteophytes. He underwent osteophytectomy through an anterolateral approach [12]. An updated systematic review published by Harlianto et al. from July 2010 to June 2021 reported that the number of cases of dysphagia had doubled in the last decade. But only 4 out of 215 patients had respiratory symptoms, and 66% (276) of patients underwent surgical treatment. The spectrum of clinical presentation included weight loss and neck pain in 62 (29%) patients, dysphonia in 55 (26%) patients, limited range of neck movement in 38 (18%) patients, sleep apnoea/snoring in 20 (9%) patients, aspiration in 20 (9%) patients, myelopathy in 20 (9%) patients, cough in 17 (8%) patients, odynophagia in 13 (6%) patients, respiratory insufficiency and aspiration pneumonia in 8 (4%), dizziness in 6 (3%) patients, choking episodes in 3 (1%) patients and radiculopathy in 2 (1%) [14].

Our case highlights the rare but significant presentation of cervical DISH with upper airway obstruction leading to stridor and respiratory compromise. Clinicians should be aware of this rare cause of acute respiratory distress.

## Conclusions

This case illustrates an uncommon but clinically significant manifestation of DISH, where excessive ossification of the cervical spine led to upper airway obstruction, resulting in stridor and respiratory compromise. While DISH is frequently asymptomatic or associated with only mild musculoskeletal symptoms, this case emphasizes that cervical spine involvement can present with life-threatening complications due to anterior osteophyte formation. These bony outgrowths can impinge upon the pharynx, larynx, or trachea, leading to airway narrowing and potentially acute respiratory failure. Timely diagnosis through appropriate imaging modalities and collaboration among medical specialists is crucial for effective management. In this case, rapid initiation of airway protection, supportive measures, and eventual surgical intervention were pivotal in stabilizing the patient and preventing further complications.

This case underscores the need for clinicians to consider DISH in the differential diagnosis of patients presenting with unexplained stridor, dysphagia, or other upper airway symptoms. Early recognition and intervention are vital to reducing morbidity and avoiding potentially fatal outcomes.
